# Summary of Proceedings Texas State Medical Association

**Published:** 1886-05

**Authors:** W. J. Bust

**Affiliations:** Sec’y. T. S. M. A.


### A SUMMARY OF THE PROCEEDINGS OF THE TEXAS STATE MEDICAL ASSOCIATION: SESSION OF 1886.

The 18th annual convocation of the Texas State Medical Association occured in Dallas, April, 27th, and continued four days. The reception committee of which Dr. R. H. Chilton was chairman, had made elaborate arrangements for the work of the Association, and for the social enjoyment of its members.

Addresses of welcome were made by Hon. John Henry Brown, Mayor of Dallas, and by Dr. S. D. Thruston on behalf of the Dallas County Medical Society, and by Hon. Seth Shepherd. Eighty-five physicians were admitted to membership.

The President, E. P. Becton, M. D., made numerous recommendations, which were referred to committees. The committee of which Dr. H. C. Ghent was chairman, made an elaborate report with resolutions, endorsing the action of the American Medical Association at New Orleans in 1885, and strongly supporting the American Medical Association in its work of organizing the Ninth International Medical Congress, and instructing the Texas delegation to vote as a unit in sustaining the American Medical Association in its laudable work.

Dr. F. E. Daniel presented a resolution which was adopted, allowing all members whose names had been dropped from the rolls on account of non-payment of dues, to be reinstated by paying the initiation fee and current dues—the same as new members.

The proposed amendment of the by-laws reducing the annual dues to $2.50 was lost.

Dr J. Larendon, treasurer, reported amount on hand April 1885, $565.12: collected for dues to April 1886, $984.50; collected for interest $52.25; total $1591.56.

Amount disbursed during the time $1546.34, leaving in the treasurer's hands $45.52. Amount collected at Dallas over $1400.

Dr. Sam R. Burroughs, Chairman of Committee on collective investigation, read an interesting and instructive report.

Dr. Geo. Cupples, Chairman of Committee on Texas Surgery, presented a synopsis of his work, showing that 138 surgeons have made 189 reports, giving in detail 4293 operations performed by Texas physicians. [See transactions. Ed.]

About forty-six papers in the various sections were read, or read by caption, and referred to the publishing committee. Some of these are reports of chairmen of sections, and others essays or reports of cases.

OFFICERS FOR 1886.

T. H. Nott, M. D., President, Goliad; R. H. Chilton, M. D., 1st. Vice President, Dallas; H. L. Parsons, M. D., 3rd Vice President, Kaufman; W. J. Burt, M. D., Secretary, Austin; J Larendon, M. D. Treasurer, Houston.

Section on Practice, etc., J. F. Y. Paine, Chairman.

On Obstetrics, etc., J. W. McLaughlin, Chairman.

On Surgery, etc., J. D. Osborn, Chairman.

On Jurisprudence, etc., F. C. Ford, Chairman.

On State Medicine, etc., R. M. Swearingen, Chairman.

On Gynæcology, F. H. Tucker, Chairman.

On Ophthalmology, etc., T. J. Tyner, Chairman.

On Dermatology and Medical Bot. H. L. Taylor, Chairman.

On Electro-Therapeutics, F. T. Paine, Chairman.

Drs. F. E. Daniel, Chairman, W. J. Burt and Q. C. Smith, of Austin, were continued as publication committee.

Austin was chosen for the place of meeting on the 4th Tuesday in April, 1887.

W. J. Bust, M. D.
Sec'y. T. S. M. A.

